# Bacterial profile and antibiotic selection for pediatric appendicitis: A retrospective cohort study

**DOI:** 10.1016/j.sopen.2023.07.018

**Published:** 2023-07-27

**Authors:** Hussein Naji, Joanna Jayakumar, Rola Ali

**Affiliations:** a Mohammed Bin Rashid University of Medicine and Health Sciences, Dubai, United Arab Emirates; b Mediclinic Parkview Hospital, Dubai, United Arab Emirates; c Health Call, United Arab Emirates

**Keywords:** Acute appendicitis, Antibiotics, Prophylaxis, Children, Pediatric, Laparoscopic appendectomy

## Abstract

**Objective:**

The objective of this study was to identify the predominant bacteria cultured from the surface of removed appendices in pediatric patients with acute appendicitis and determine the appropriate choice of antibiotics for preoperative and postoperative management.

**Methods:**

A 2-year retrospective cohort study was conducted at Mediclinic Parkview Hospital, Dubai, UAE. Patients under 14 years of age with diagnosed acute appendicitis who underwent laparoscopic appendectomy were included. Swab cultures, along with demographic, laboratory, and pathology data, were analyzed.

**Results:**

Out of the 56 enrolled patients, 27 (48 %) exhibited bacterial growth on swab cultures, while 29 (52 %) showed no bacterial growth. *Escherichia coli* (*E. coli*) was the predominant isolated bacteria, present in 23/27 patients (85 %). Seven patients had co-infections involving *E. coli* and other bacteria, with Pseudomonas being the second most common bacteria identified in 7/27 patients (25 %). Antibiotic sensitivity testing indicated that 85 % of the isolated bacteria were sensitive to Gentamicin, 63 % to Amoxicillin/Clavulanic acid, 59 % to Trimethoprim + Sulfamethoxazole, and 27 % to Cefazolin.

**Conclusion:**

*E. coli* was the most prevalent bacteria identified on swabs taken from inflamed appendices in pediatric patients. Amoxicillin/Clavulanic acid was determined to be an appropriate choice for preoperative prophylaxis. This study provides valuable insights for guiding the management of pediatric appendicitis and facilitating the appropriate and judicious use of antibiotics.

## Introduction

Appendicitis is a frequently encountered emergency condition in the pediatric population, affecting both males and females with a lifetime risk of 8.6 % and 6.7 %, respectively [1,2]. In the United States of America, it ranks as the fifth most common cause for pediatric hospital admissions, with a rate of 97.4 stays per 100,000 admissions [1,2]. The incidence of acute pediatric appendicitis in the 21st century varies geographically, ranging between 100 and 151 cases per 100,000 person per year [3].

While appendectomy remains the primary approach for managing acute appendicitis in most pediatric patients, there are certain situations where non-operative management with antibiotics presents a viable alternative [[4], [5], [6], [7]]. Despite advancements in aseptic practices and surgical techniques, postoperative complications such as wound infections and intra-abdominal abscesses still contribute to significant morbidity. Previous studies have suggested that administration of antibiotic regimens can help reduce the incidence of postoperative infections, leading to improved outcomes and decreased morbidity and mortality rates [8]. Additionally, the utilization of antibiotics as preoperative prophylaxis has shown promising results, including reduced hospital stays per patient and overall healthcare expenses [9].

The objective of this study is to enrich global databases by identifying the predominant bacteria that can be isolated from the surfaces of removed appendices, along with determining the appropriate antibiotics for their treatment. By providing insights into optimal antibiotic choices, this research aims to support antibiotic stewardship programs in reducing unnecessary antibiotic usage, thereby facilitating effective treatment of appendicitis alongside laparoscopic appendectomy and minimizing the risk of postoperative complications.

## Materials and methods

### Study design

This retrospective cohort study was conducted over a period of 2 years at Mediclinic Parkview Hospital in Dubai, United Arab Emirates. The study aimed to investigate the management of acute appendicitis in children under 14 years of age. Ethical approval was obtained from Mediclinic Middle East Research and Ethics Committee (MCME.CR 150. MPAR. 2020) and Dubai Scientific Research Ethics Committee (DSREC), DHA (DSREC-11/2020_18).

### Inclusion criteria

The study included all children under the age of 14 years who were diagnosed with acute appendicitis and underwent laparoscopic appendectomy at Mediclinic Parkview Hospital from September 2018 to August 2020. The diagnosis of acute appendicitis was based on clinical evaluation and radiological investigations.

### Variables analyzed

The variables analyzed in this study included age, gender, laboratory investigations, and pathology reports of the removed appendices. Pathology reports were reviewed to determine the presence of various pathological findings.

### Swab culture and sensitivity

During the operative procedure, a swab was taken from the surface of each removed appendix. These swabs were sent for culture and sensitivity testing to identify bacterial growth and determine antibiotic sensitivity.

### Exclusion criteria

Eight patients who were diagnosed with acute appendicitis during the peak of the COVID-19 pandemic outbreak (March–June 2020) and were treated with only medical treatment using antibiotics were excluded from the study. They did not undergo surgical intervention due to restrictions imposed to prevent the spread of the pandemic.

### Statistical analysis

Data analysis was performed using the Statistical Package for the Social Sciences (SPSS) version 24. Descriptive statistics were used to summarize the data, and appropriate statistical tests were applied to analyze the findings.

## Results

A total of 56 patients underwent appendectomy and were included in the study. Among them, 18 patients (32 %) were female, and 38 patients (68 %) were male. The age of the patients ranged from 4 to 14 years, with a mean age of 9 years. The distribution of age and gender is summarized in Table 1.

**Table 1:** Age and gender distribution of the included patients.

Age	Male	Female	Total	Percentage
4–7	7	7	14	25
8–11	23	7	30	53.6
12–14	8	4	12	21.4
Total	38	18	56	100

Pathology reports were reviewed for all 56 resected appendices, confirming the presence of acute appendicitis in every case. The following pathological findings were observed: perforation in 39 % (22/56) of the patients, gangrenous appendix in 18 % (10/56) of the patients, eosinophilic infiltration in 1.8 % (1/56) of the patients, and the presence of intestinal worms in 1.8 % (1/56) of the patients.

**Fig. 1:**
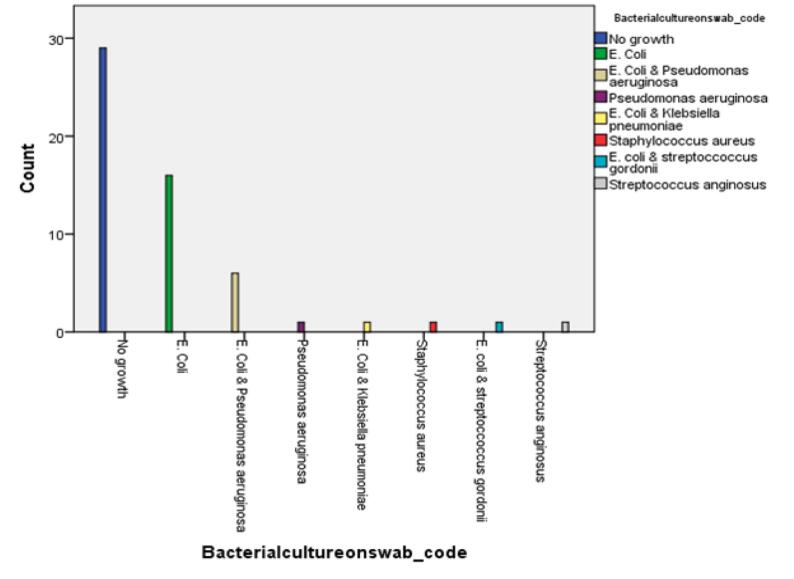
Prevalence of Bacterial types isolated from the removed appendices.

Swab cultures were performed on the removed appendices, and bacterial growth was observed in 27 out of 56 patients (48 %). The most common isolated bacteria from the inflamed appendices were *Escherichia coli* (*E. coli*), found in 23 patients (85 %), out of which 7 patients had a co-infection with other bacteria. Pseudomonas bacterial infection was identified in 7 patients (25 %), with 5 of them having a co-infection. Additionally, *Klebsiella pneumoniae* (3 %), *Staphylococcus aureus* (3 %), *Streptococcus gordonii* (3 %), and *Streptococcus anginosus* (3 %) were also identified as isolated bacteria. There was no significant difference in the isolated bacteria between perforated and non-perforated appendices. The prevalence of bacterial types isolated from the removed appendices is illustrated in Fig. 1.

Antibiotic sensitivity testing showed that 85 % of the isolated bacteria were sensitive to Gentamicin, 63 % to Amoxicillin/Clavulanic acid, 59 % to Trimethoprim/Sulfamethoxazole, and 27 % to Cefazolin.

All patients enrolled in the study received routine prophylactic preoperative antibiotics. Amoxicillin/Clavulanic acid was administered intravenously, 30 min prior to surgery, according to the study protocol, and it was used in 54 patients (96 %). Two patients who were allergic to penicillin received Ceftriaxone and Metronidazole as an alternative to Amoxicillin/Clavulanic acid. If there is no perforation, the antibiotics are stopped. If there is perforation or gangrenous appendicitis, the antibiotics are continued postoperatively for 5 days.

Infectious complications after appendectomy were encountered in 4 patients in the form of intra-abdominal collection of fluid that required additional IV antibiotic therapy. One of them developed sepsis and needed 5 days stay in hospital. One patient had a suprapubic wound dehiscence, and 2 patients had a yellowish discharge from the sub-umbilical wound. One (1.8 %) patient needed to be readmitted to the hospital for additional IV antibiotic treatment post-surgery because of an abdominal abscess. All patients were successfully managed and eventually discharged from the hospital.

## Discussion

The use of antibiotic prophylaxis in children undergoing appendectomy remains a topic of ongoing discussion. While the effectiveness of antibiotic prophylaxis in reducing postoperative infections is well established, concerns regarding antibiotic resistance development and associated adverse effects persist. A comprehensive Cochrane review involving 45 studies and 9576 patients demonstrated that antibiotics were superior to placebo in preventing wound infection and intra-abdominal abscesses [8].

Notably, the Infectious Diseases Society of America, the Surgical Infection Society, and the Society for Healthcare Epidemiology of America recommend broad-spectrum antibiotic prophylaxis for acute appendicitis, whether employing single or combination agents [8]. Conversely, the local and national protocols for Empirical Antibiotic Prescribing for Children in the United Kingdom suggest IV Amoxicillin + IV Gentamicin + IV Metronidazole [9]. In our clinical practice, we have utilized amoxicillin/clavulanic acid in patients without penicillin allergy and ceftriaxone in those with a penicillin allergy.

Considering these diverse guidelines, we recognized the need for a clinical pathway to aid physicians in the empirical management of preoperative and postoperative appendectomy for acute appendicitis in children. To construct an evidence-based pathway, we analyzed the bacterial prevalence derived from intraoperative swab cultures obtained from removed appendices. Our study identified bacterial growth in 48 % of the swabs, with *E. coli* being the most common isolate, either alone or in combination with other bacteria, accounting for 85 % of positive growth. Additionally, our findings revealed that Gentamicin exhibited sensitivity against most isolated bacteria (85 %). However, the known risk of ototoxicity and nephrotoxicity associated with Gentamicin [[10], [11], [12]] warrants close monitoring of renal function. Considering the potential side effects and the risk of promoting antibiotic-resistant bacteria, Gentamicin is not an ideal choice for preoperative prophylaxis in children.

Furthermore, our study demonstrated that 63 % of the cultures with bacterial growth were susceptible to amoxicillin/clavulanic acid. This antibiotic is well-tolerated, cost-effective, and commonly prescribed in children due to its broad-spectrum activity against various bacteria [13,14]. It combines amoxicillin, which targets a wide range of bacteria including streptococci, pneumococci, staphylococci, and *E. coli*, with clavulanic acid, a beta-lactamase inhibitor that expands its effectiveness against bacteria resistant to amoxicillin alone. Amoxicillin/clavulanic acid provides adequate coverage against anaerobic species, such as *Bacteroides fragilis* and Prevotella species [15], making it a suitable choice for surgical prophylaxis in appendicitis. Notably, previous research has demonstrated that amoxicillin/clavulanic acid alone is as effective as the metronidazole/gentamicin combination in preventing wound infections following appendectomy [13]. Our study found that the infectious complications observed in our patients after appendectomy were comparable to those reported in the referenced studies.

We acknowledge that observational studies have inherent limitations, and drawing robust conclusions solely based on the data available can be challenging. However, we aimed to present our findings cautiously, being mindful of the study's design and limitations.

In summary, this study offers valuable insights to support clinical decision-making regarding the use of antibiotic prophylaxis in children undergoing laparoscopic appendectomy. Nevertheless, further research is necessary to validate these findings and explore the optimal duration and selection of prophylactic antibiotics for this patient population.

## Conclusion

Our study serves as a valuable resource for guiding the management of pediatric appendicitis and promoting the appropriate and careful use of antibiotics. The predominant bacterial growth observed in the appendiceal swabs was attributed to *E. coli*. Notably, our findings support the effectiveness of amoxicillin/clavulanic acid as a suitable option for perioperative prophylaxis when used as a single antibiotic. However, further research is warranted to validate these results and explore the optimal duration of treatment in this context. By expanding our knowledge in these areas, we can enhance patient care and optimize treatment strategies for pediatric appendicitis.

## Funding

This research received no specific grant from any funding agency.

## Ethical approval

The study was approved by the Mediclinic Middle East Research and Ethics Committee (MCME.CR 150. MPAR. 2020) and Dubai Scientific Research Ethics Committee (DSREC), DHA (DSREC-11/2020_18).

## Consent for publication

All of authors were consented.

## CRediT authorship contribution statement

Hussein Naji: conception and design, analysis and interpretation, data collection, writing the article and critical revision of the article.

Joanna Jayakumar: data collection, analysis and interpretation and writing the article.

Rola Ali: analysis and interpretation, writing the article and critical revision of the article.

## Declaration of competing interest

The authors declare no conflict of interest.

## Data Availability

The data sets used and/or analyzed during the current study are available from the corresponding author on reasonable request.
